# Maghrebi minors as translators in health services in Tarragona (Spain): a qualitative study of the discourse of the Maghrebi adults

**DOI:** 10.1186/1744-8603-10-31

**Published:** 2014-05-07

**Authors:** Lourdes Rubio-Rico, Alba Roca Biosca, Inmaculada de Molina Fernández, M Mercè Viladrich Grau

**Affiliations:** 1 Department of Nursing, Rovira i Virgili University, Tarragona, Avda Catalunya, 35, 43002 Tarragona, Spain; 2 Unesco Intercultural Dialogue Chair, Rovira i Virgili University, Avda Catalunya, 35, 43002 Tarragona, Spain; 3 Department of Semitic Philology, Faculty of Philology, Edifici Històric, University of Barcelona, 1ª planta, Gran Via de les Corts Catalanes, 585, 08007 Barcelona, Spain

**Keywords:** Immigrants, Africa –Northern, Child, Minors, Child advocacy, Translating, Language barriers, Cultural diversity

## Abstract

**Background:**

In the province of Tarragona (Spain), 24% of immigrants come from countries in the Maghreb. 40% of Maghrebis residing in Spain say their linguistic command of Spanish is inadequate, which could hinder their relationship with healthcare professionals. The use of minors as translators by health services is a fairly common practice. The suitability of using children as translators has been questioned, although there has been little specific research on the subject and most has been from the perspective of professionals. The aim of this study was to qualitatively analyze the discourse of Maghrebi adults to the use of Maghrebi minors as translators in the health services.

**Methods:**

A qualitative study using 12 in-depth interviews and 10 focus groups with Maghrebi adults living in Tarragona. The scope of the study was primary healthcare and hospital services in the area. A content analysis was performed using open coding.

**Results:**

The practice studied is attributed to a lack of funding for translation resources, and prioritization of adults’ work over children’s education. It is seen as a convenient solution to the community’s communication problems, although it is considered unreliable and detrimental to the rights of the child. The attitudes of healthcare professionals to the phenomenon studied varies from acceptance without any ethical concerns to concern about its effects on the child. The solutions proposed are the organization of translation resources with a proactive approach which are adapted to real needs, and a change in the focus of language training activities for the adults in the community.

**Conclusions:**

It is necessary to reconcile access to healthcare for Maghrebi adults with the rights of children who act as translators in the healthcare context. This requires coordination between health and educational institutions, changes in the organization and provision of translation resources, and a guarantee that immigrants have employment rights under the same conditions as Spanish nationals.

## Background

In 2009, when the fieldwork for this article was carried out, the Spanish public health system guaranteed care for foreigners under the same conditions as Spaniards, regardless of their legal status. The only enforceable requirement was a relatively simple process whereby foreigners had to register as residents with the local authority concerned, although this requirement was not applied to children and pregnant women, who still enjoyed full coverage irrespective of their legal and administrative situation [1].

According to figures from the Spanish National Statistics Institute [2] the percentage of foreign-born residents among the total residents in Spain on 1 January 2011 was 14%. Of these, 12% were from the Maghreb–mainly from Morocco and Algeria, which are the countries of origin of 99.7% of the Maghrebis living in Spain come from. In the province of Tarragona (Spain), where this study was conducted, foreign-born inhabitants account for 19% of the population, of which 24% is of Maghrebi origin.

Data form the 2007 National Migration Survey [2] (compiled by the authors) shows that 40% of Maghrebis residing in Spain say that their linguistic command of Spanish is inadequate. The same source states that the percentage of Maghrebi women who acknowledge having language difficulties is 54%, compared to 33% of men in the same situation. In this context, there is some agreement that language difficulties are a major barrier to accessing and using health services [3-5].

The need for translators in the health field has been recognized by several studies [3,6,7], and healthcare institutions have therefore considered strategies to solve the problem [7]. The resources that healthcare institutions place at the disposal of users with an inadequate linguistic command include telephone translation services, intercultural health mediators and reference materials in various formats [5,7-9]. The role of intercultural health mediators is to facilitate communication between professionals and users by means of linguistic and cultural translation and interpretation. Telephone interpreting and reference materials are intended as services that complement face-to-face interpretation, when the latter is not available or is beyond the capacity of the intercultural health mediator for specific reasons [10].

In the healthcare field, the advantages of translation by professional experts have been demonstrated in comparison to other alternatives such as family members, including children. Translation by professional experts improves communication, user satisfaction and health outcomes, and reduces interpretation errors [11-15].

Translation in healthcare guided by children affects the quality of the interaction due to the emotional involvement of the translator–the child–who ceases to be impartial, as would be desirable [11,16-19]. Strictly linguistic difficulties such as limitations in vocabulary when translating parts of the body and medical terminology, and the lack of equivalence of some words or expressions across the two languages, makes translation and comprehension extremely difficult [16-18]. The child’s curtailment of the message when discussing taboo subjects or functions means that information that could be decisive is not passed on [11,16,18], and translation undertaken by children places excessive responsibility on the children concerned and forces professionals to take hazardous decisions, in that they are perhaps based on erroneous information [16-18].

Translation undertaken by minors does not only affect the quality of communication, but also affects the children involved. This effect takes the form of shame when discussing certain subjects, the emotional impact involved in having full knowledge of adults’ illnesses, the obligation to assume certain responsibilities prematurely, the inversion of the family hierarchy, and the eschewal of some children’s activities by those involved [15,16,18,20].

The use of minors as translators in health services is fairly common practice among general practitioners [16]. In the United States, 22% of professionals who use interpreters to overcome language barriers in the surgery use children [21]. These figures give an idea of the level of tolerance of professionals and users toward a phenomenon that can at least be considered irregular. Despite no data quantifying the phenomenon in Spain being available, translation by children is accepted–not without hesitation–as one of the resources available to overcome language barriers in the health services [9,22].

The suitability of using children as translators is often called into question in the manuals for treating people with a poor command of language [11,19], although there has been little specific research on the subject, and most of it considers the issue from the perspective of professionals [15-17,23]. Moreover, the causes of the phenomenon have not been studied in depth and no suggestions for improvement have been made.

This paper presents part of the results of another broader study, that aimed to understand the discourse of Maghrebi adults regarding the use of Maghrebi minors as translators in the health services in the province of Tarragona.

## Methods

### Design

We used a qualitative design for descriptive and interpretive purposes. The choice of this type of design is justified by the need to ascertain the assessments, causal attributions, the effects felt and proposals for improvement among Maghrebi adults as regards the use of minors for translation in the health services, in order to contribute to raising awareness of the problem and finding a solution to it.

This study considered *minors* to be children under 16 years old, since that is the age threshold for compulsory education in Spain.

The data were collected in semi-structured interviews and focus group sessions. The scope of the study was primary healthcare and hospital services in the province of Tarragona.

### Participants

The study population was considered to be adults (≥18 years) born in any of the countries of the Maghreb, residing in the province of Tarragona and with recent experience (≤6 months) of the health services in the area, both in primary care and hospital. The study population was not required to have participated directly in translation scenarios guided by children because it was considered that merely by being members of the community studied they would be able to offer their views on the subject.

Purposive sampling was conducted until data saturation was achieved. The initial aim was to organize focus groups of adult users based on the information providers’ gender, cultural affiliation (Arab or Berber), level of education, language proficiency and legal situation. It was not possible to organize an entirely male focus group. The men who participated in the study did so in mixed groups or by means of individual interviews. We also found it difficult to recruit users who agreed to the data about their legal status being recorded. We therefore decided not to organize focus groups based on this characteristic, and decided to ensure the presence of information providers who had been in Spain for less than one year in the overall participants. To complete the spectrum of people with specific difficulties in relation to health services, we decided to include the testimony of two people who were not registered as residents and therefore had no access to the public health care system, despite having expressed the need to use it.

As the data collection progressed, and in view of the need to complement the user’s limited experience with another that was more extensive and comprehensive, the testimony of Maghrebi adults who were working as intercultural health mediators for the studied group was included in the research. The treatment of women as health agents par excellence among the Maghrebi community [24] suggested that an overrepresentation of women would be appropriate. Data saturation was accepted when new categories stopped appearing in the coding [25].

Access to the Maghrebi adults took place in the community. The recruitment of information providers took place using the snowball technique, with people of Maghrebi origin initially accessible to the researcher, or through health and social work professionals who were in contact with the community, who were able to recruit individuals or groups of people from the study population. The professionals who acted as intermediaries for contacting the information providers included nurses, social workers, immigration experts, cultural health mediators, and volunteers in community outreach work. Some of these professionals contacted the information providers in a personal capacity and others did so on behalf of the institutions for which they worked. All the individuals and institutions who assisted in the recruitment of participants agreed to the study protocol beforehand, and gave their approval.

The data were collected between May 2009 and May 2011 by means of semi-structured interviews and the organization of focus groups. A pilot test was conducted in the first two focus group sessions, after which it was decided to include an external translator in all sessions with users and to complete the oral consent with an informed consent form in Arabic that was produced starting from this point.

A script with references to key issues was followed in both the individual interviews and the focus group sessions. Each of the key issues was associated with a series of trigger questions as an example, in order to begin and lead the discussion and interviews. Additional file 1: Table S1 shows the subjects covered in the original study and the trigger questions associated with the use of Maghrebi minors as translators in the health services.

When assigning data collection techniques to the type of respondents, we decided that the mediators should be interviewed individually to gain an in-depth overview of the subject for study. The focus group technique was decided upon for users, and to foster discussion. Some users expressed their reluctance to participate in focus groups and others had difficulties in terms of time availability to attend the meeting. In both cases, they were offered the opportunity to participate in the study by means of an individual interview.

The individual interviews were conducted at a venue proposed by the respondent. The focus group sessions took place in neutral spaces agreed upon by both parties or in family spaces for the participants, since many of the focus groups were groups that had previously been organized for other reasons (literacy, neighbourhood, university, etc.). All the data collection activities were conducted by the same interviewer (LR-R). Sessions with information providers who were not competent in either of the area’s official languages (Spanish or Catalan) used an Arabic or Tamazight translator, depending on the language of expression of the group or individual. From the third focus group session onwards, the translator was a person who did not belong to the discussion group. The sessions were recorded in audio, and a literal transcript of the translated content was made.

12 individual interviews were conducted–6 with mediators and 6 with users, one of whom had acted as a translating minor. 10 focus groups of 3 to 11 participants were organized and had the characteristics shown in Table 1.

**Table 1:** Description of focus groups

**ID. GROUP**	**Segmentation**	**Number of participants**	**Duration**	**Sex**	**Age:**	**Linguistic command:**	**Cultural affiliation:**	**Environment of origin:**	**Educational level:**
					**Mean (min - max)**	**N (%)**	**N (%)**	**N (%)**	**N (%)**
FG1	Women. Arab. Poor linguistic command.	6	44’	Women: 6 (100%)	34 (21–55)	None or low: 6 (100%)	Arab: 6 (100%)	Urban: 5 (83%)	Illiterate: 2 (33.3%)
								Rural: 1 (17%)	Literate: 1 (16.7%)
									Primary ed.: 1 (16.7%)
									Secondary ed.: 2 (33.3%)
FG2	Women. Arab. University	3	137’	Women: 3 (100%)	30 (25–37)	Basic: 2 (66.7%)	Arab: 3	Urban: 2 (67%)	Higher: 3 (100%)
						High: 1 (33.3%)	(100%)	Rural: 1 (32%)	
FG3	Women. Berber. Primary education not completed. Poor linguistic command. Rural environment.	7	77’	Women: 7 (100%)	36 (27–42)	None or low: 7 (100%)	Berber: 7 (100%)	Rural: 7 (100%)	Illiterate: 3 (42.9%)
									Literate: 4 (57.1%)
FG4	Women. Arab. Poor linguistic command.	5	46’	Women: 5 (100%)	35 (27–41)	None or low: 5 (100%)	Arab: 5 (100%)	Urban: 1 (20%)	Illiterate: 2 (40%)
								Rural: 4 (80%)	Literate: 2 (40%)
									Secondary ed.: 1 (20%)
FG5	Women. Berber. Poor linguistic command.	4	58’	Women: 4 (100%)	39.50 (28–55)	None or low: 4 (100%)	Berber: 4 (100%)	Urban: 3 (75%)	Illiterate: 3 (75%)
								Rural: 1 (25%)	Secondary ed.: 1 (25%)
FG 6	Women. Arab. Primary and secondary education completed	5	55’	Women: 5 (100%)	28 (19–43)	None or low: 1 (20%)	Arab: 5 (100%)	Urban: 4 (80%)	Primary ed.: 2 (40%)
						Basic: 4 (80%)		Rural: 1 (20%)	Secondary ed.: 3 (60%)
FG7	Women. Arab. Primary and secondary education completed. Urban environment.	3	52’	Women: 3 (100%)	28 (27–32)	None or low: 2 (66.7%)	Arab: 3 (100%)	Urban: 3 (100%)	Primary ed.: 2 (66.7%)
						Basic: 1 (33.3%)			Secondary ed.: 1 (33.3%)
FG8	Mixed. Poor linguistic command. Urban environment.	9	91’	Women: 6 (66.7%)	26 (22–37)	Basic: 9 (100%)	Arab: 5 (55.6%)	Urban: 9 (100%)	Primary ed.: 3 (33.3%)
				Men: 3 (33.3%)			Berber: 4 (44.4%)		Secondary ed.: 3 (33.3%)
									Higher: 3 (33.3%)
FG9	Mixed. Poor linguistic command. Urban environment.	4	96’	Women: 1 (25%)	28.5 (24–45)	Basic: 4 (100%)	Arab: 3 (75%)	Urban: 4 (100%)	Primary ed.: 1 (25%)
				Men: 3 (75%)			Berber: 1 (25%)		Secondary ed.: 2 (50%)
									Higher: 1 (25%)
FG10	Women. Poor linguistic command.	11	67’	Women: 11 (100%)	43 (24–52)	None or low: 11 (100%)	Arab: 6 (54.5%)	Urban: 10 (91%)	Illiterate: 8 (72.7%)
							Berber: 5 (45.5%)	Rural: 1 (9%)	Primary ed.: 1 (9.1%)
									Secondary ed.: 2 (18.2)

### Ethical considerations

Although this project did not focus on any healthcare institution and did not recruit any patients, but instead adults with experience as users of health services or with a perceived need to use that service, all the collaborating institutions had prior access to the project and gave their approval. Furthermore, to comply with the ethical requirements of research, an informed consent document was produced. This document listed the objectives of the study, the data collection techniques, the author’s affiliation, the voluntary nature of participation and the possibility of withdrawal from the study. Confidentiality, the information providers’ anonymity and the use of information solely for research purposes were also guaranteed. This document was translated into Arabic in a sworn translation and from the point when it became available (February 2010), it was accepted and signed by all participants able to read and write in that language. The participants in the study who were not literate in Arabic or who had provided testimony before the consent document was available gave their oral consent after being informed of the same aspects as those listed in the written consent.

### Data analysis

An analysis of the content was performed. The first level of analysis was concurrent with the data collection and aimed to identify emerging themes to modify the sampling and data collection work. In the second level, the data were segmented and the reporting units were identified, and coded using open coding. The categories that emerged were integrated into a higher level of organization based on the properties and dimensions of a single concept. The interpretation of the data sought to establish relationships between different levels of organization of the content, either between categories, between concepts or between categories and concepts. These processes were carried out by the lead author of the article.

To ensure the impartiality of the lead author’s analysis, we decided to submit it to two healthcare professionals with experience in treating Maghrebi users, and another non-healthcare professional who was an expert in Islamic culture so that they could determine its validity. The units of analysis to be verified were organized in accordance with the topics that emerged from the data. Each of the topics was considered twice to determine its validity. First of all, the researchers considered, one by one, the validity of the analysis made by the lead researcher. Secondly, any discrepancies were discussed and resolved in a meeting between the four researchers involved in the analysis.

The systematization of the categorization and analysis was performed using the program *ATLAS-ti* WIN version 5.0.

## Results

The sociodemographic characteristics of the participants are shown in Table 2.

**Table 2:** Sociodemographic characteristics of respondents

	**Users**		**Mediators**		**Total**	
Age in years: mean (CI 95%)	35 (33–38)		39 (30–47)		36 (33–38)	
Duration of the interview in minutes: mean (CI 95%)	69' (63' - 75')		76' (57' - 95')		70' (64' - 75')	
	n	%	n	%	n	%
Man	9	14.3%	1	16.7%	10	14.5%
Woman	54	85.7%	5	83.3%	59	85.5%
Year of arrival						
Before 1995	2	3.2%	0	0.0%	2	2.9%
1995-1997	6	9.5%	1	16.7%	7	10.1%
1998-2000	5	7.9%	0	0.0%	5	7.2%
2001-2003	13	20.6%	2	33.3%	15	21.7%
2004-2006	18	28.6%	3	50.0%	21	30.4%
2007-2009	14	22.2%	0	0.0%	14	20.3%
2010	5	7.9%	0	0.0%	5	7.2%
Type of interview						
Focus group	57	90.5%	0	0.0%	57	82.6%
Individual	6	9.5%	6	100.0%	12	17.4%
Cultural affiliation						
Arab	39	61.9%	5	83.3%	44	63.8%
Berber	24	38.1%	1	16.7%	25	36.2%
Environment of origin						
Rural	21	33%	2	33%	23	33%
Urban	42	67%	4	67%	46	67%
Linguistic command						
None or low	41	65.1%	0	0.0%	41	59.4%
Basic	19	30.2%	0	0.0%	19	27.5%
Advanced	3	4.8%	6	100.0%	9	13.0%
Level of education						
Unable to read or write	20	31.7%	0	0.0%	20	29.0%
Able to read and write	7	11.1%	0	0.0%	7	10.1%
Primary educ.	13	20.6%	0	0.0%	13	18.8%
Secondary ed.	15	23.8%	1	16.7%	16	23.2%
University	8	12.7%	5	83.3%	13	18.8%
Experience of care						
Primary and hospital	55	87.3%	5	83.3%	60	87.0%
Primary	6	9.5%	1	16.7%	7	10.1%
Perceived need	2	3.2%	0	0.0%	2	2.9%

The statements on this topic were organized into five key areas: *The causes, Perception of professionals’ attitudes, Attitude of Maghrebi adults, The consequences* and *Proposals for improvement*. A summary of the main arguments related to the five key areas is shown in Table 3.

**Table 3:** Summary of the results of the key points

**Causes**	Poor language proficiency among Maghrebis
	Very limited access to translation/mediation resources
	Translation/mediation services not proactively organized
	Maghrebi adults prioritiz their employment responsibilities over children’s education
**Perceived attitudes among health professionals**	Positive attitude to using children as translators in health services
	No desire to encourage the participation of children, but no opposition to it
	Opposition to the practice for several reasons (quality of translation and perpetuation of dependence effect among users)
	Opposition to the practice because it infringes on the child’s rights
**Maghrebi adults’ attitudes**	Willingness to routinely use support resources
	Willingness to occasionally use support resources (but not in situations where sexual or reproductive issues are discussed, or where this would lead to missing school)
	A belief that children should not used in this way
**Consequences**	Translations are of poor quality
	The children miss school
	Children are emotionally affected
	Family hierarchies are inverted
	Child’s self-esteem may be affected positively
**Proposals for improvement**	Collaboration between educational and health care institutions Promotion of firm attitudes against using children as translators
	Provision of sufficient mediation/translation services
	Promotion of language learning among Maghrebi immigrant
	Provision of the same employment rights for immigrants and Spanish nationals

### The causes

Given their difficulties with expressing themselves in Spanish, Maghrebi users often need the help of a translator to communicate with the healthcare team: “*She always goes with a translator, either a friend, a sister, a neighbour, the children…*” (FG10. Women. Poor linguistic command).

The adults interviewed believe that there is very limited support for intercultural health mediators: “*We always work with grants* […]. *If there are no grants, you don’t work*” (II11. Mediator. Women) and lack professionals and service hours, leading to a mismatch between the institutional supply of mediators and the translation needs of users from the community, who are forced to resort to translators, including children, on an *ad hoc* basis: “*I’m only at the primary healthcare centre* [PHC] *on two days, and I’m not there in the afternoon … and sometimes.. children… will have to go in with their mother, but of course, there is no alternative. There is no other solution to do the translation*”. (II9. Mediator. Woman).

As well as the mismatch between the demand for translation and the supply of intercultural health mediators, there is a lack of proactivity in the organization of some mediation services. According to the information providers, the mediator is not part of the health team. Together with a limited promotion of the service among professionals, this means that they are underused. In these circumstances, situations requiring a mediator may arise and be resolved without their presence, despite the service being available: “*Mediators exist, you know! It’s not that there isn’t a mediator, there is one and they don’t call them* […]. *Until people get the idea that there’s a mediator, they think that there isn’t one. A mediator, well, should be part of the health team*” (II11. Mediator. Woman). Alternatively, users may become aware of the existence of the mediation service by alternative means, with the consequent loss of opportunities to use the service: “[…] *They haven’t said anything to him* [about the mediation service]*, instead through his friend, he has come to ask* […] *if there is someone who works as a mediator here at the hospital, here in the surgery”* (II5. User. Man).

Finally, the prioritization of employment responsibilities over children’s education, and the minors’ sense of obligation to their parents, transfers the responsibility for support and translation from linguistically competent adults to children: “*Because … there are many children who miss school. And sometimes your parents force you, you can’t say that you won’t go with your father or mother* […]*. Because the man, what with work and everything, usually tries not to miss it, so… it’s like we were saying, they don’t value education so much* […] *if I miss school it’s okay, but them … you know? Because they don’t pay you at school and they do at work*” (II7. User. She translated when she was a child).

### Perception of professionals’ attitudes

The participants in the study highlight different attitudes among the professionals dealing with situations involving children acting as translators. According to the information providers, some professionals encourage the presence of minors in appointments without considering ethical issues relating to the rights of the child or the quality of care provided under these circumstances: “*Sometimes, at first, I used to introduce myself: “Hello, I’m the mediator. I’m a health worker, I act as a bridge for mediation between professionals and patient” and she* [the professional] *says: “No, she’s come with her boy, her son… she already has a daughter that talks!”* (II9. Mediator. Woman).

The information providers identify another group of professionals which while they do not encourage the participation of children during the appointment at the surgery, they do not oppose it: “*When I was at the surgery, the doctor says to the girl: “Have you missed school?” And she says ‘yes’, and that’s it. She didn’t say that she shouldn’t… no …*” (FG9. Mixed. Poor linguistic command).

According to the information providers, there are also those who oppose the practice for various reasons, including the quality of the translation that a child can offer: “*Yes,* [professionals] *don’t want children.* […] *She says that some believe that children don’t talk about anything that …they can see that children don’t translate everything* (FG10. Women. Poor linguistic command), and those with a greater commitment to the community, who oppose translation by minors because it perpetuates the dependence effect among users: “*Some doctors told me “that women should come alone, because if they ever have to come alone they sort it out, they try to make the effort and speak for themselves, but if you’re there, they relax and you’ll do the talking”*. (II7. User. She translated when she was a child).

Finally, there are professionals who are concerned about interference in the child’s right to education, as they are forced into school absenteeism: “[the doctor] *tells them that they have to talk themselves, that they have to try to make themselves understood as much as they can, because children can’t miss school, it’s important for them to go to school*.” (FG5. Women. Berber).

From their dual perspective of health professionals and members of the community, health mediators believe that minors who translate are not linguistically or emotionally ready to deal with adult situations: “[…] *To what extent can they translate? There are specific terms, medical terms, diagnoses …* […] *How can a child translate that his mother is pregnant with a Down syndrome child? […] It’s almost impossible for a child to explain that*” (II9. Mediator. Woman). They even think that their work as translators in the health services may entail risks for the child’s health: “*Yes, yes, I’ve seen a lot* [of children] *and I, for example, say to the parents that it isn’t good for the child’s health, their mental health*” (II10. Mediator. Woman).

However, they admit that today, it is inevitable as long as the supply of translation resources does not meet the real demand for the service among the community. “*What’s more, the mediator is not there all the time that people are. And you can’t tell them not to bring their children, because you’re here today and gone tomorrow*” (II11. Mediator. Woman).

### Attitude of Maghrebi adults

The use of school age children as translators in a health context is mentioned as a translation and support resource which is routinely used: “*She has said that they look for a boy or girl to go with them. Someone who … they usually do it that way–even if it’s that girl, for example. She’s in the fifth year and often has to miss* [school] *and accompanies them*” (FG5. Women. Berber). However, there are some parents who avoid translation by minors. Some avoid it because they think that their children are still too young to translate: “[…] *her husband works, her children are young, so … Who should she ask to come and translate for her*? (FG7. Women. Primary and secondary education completed) and others because they are determined not to do it: “*And as far as children are concerned, she prefers not to bring her children to the appointment with her*” (FG7. Women. Primary and secondary education completed). As for the circumstances of the health appointment, most tend not to use children as interpreters in situations in which sexual or reproductive issues are discussed: “*That lady, what she does, when she has a routine thing … when she has a headache or something, she brings her daughter. When she has to visit the gynaecologists, she brings her husband*” (FG10. Women. Poor linguistic command).

In an attempt to minimize the consequences of using children as translators, parents try to make appointments based on the child’s availability and thereby avoid school absenteeism: “*She says she tries to make sure that they don’t miss school because of bringing them, so she sees that they aren’t studying from such-and-such a time to such-and-such a time and she makes the appointment then, she comes with her. If* [the appointment] *is in school hours, her school hours, she prefers to find a neighbour or to come alone*” (FG7. Women. Primary and secondary education completed). However, if necessary, the employment obligations of the head of the household take priority over their children’s education and the minor performs the role of accompaniment that the adult should perform: “*It was up to me to go to the doctor with my mother. And I said to my father: “Why don’t you go?” And he used to say to me: “No, because I’m the one who brings home the money*”. (II7. User. She translated when she was a child).

Finally, because of the gender implications of care and reproduction among Maghrebis, the tasks of support and translation in the health services are considered work for girls: “*Usually, it’s the girl who accompanies the doctor and everything. She… has this responsibility, not like him, not like boys, because sometimes they talk about more delicate parts, subjects…*” (II7. User. She translated when she was a child).

### The consequences

The effects of translation by minors is apparent in the quality of the interactions between professionals and users. In these circumstances, translations may lack interpretation, because children have difficulty conveying more subtle aspects of communication such as cultural and nonverbal aspects: “*Perhaps a child can convey the wrong message*” (II8. Mediator. Man).

Ignorance of the terminology involved in the doctor’s appointment makes translation difficult: “*Because before, I didn’t know what all the internal organs were, and even less in Moroccan, you know? “It’s that red thing that lambs have’, I said when explaining what the liver is*”” (II7. User. She translated when she was a child). And the refusal of the child to translate certain subjects issues may even lead to an absence of translation: “*The doctor’s message changes. Yes, because they’re embarrassed. Well … they don’t say anything about … when* [the doctor] *says “ask your mother what…” they don’t mention anything, they say nothing* (FG10. Women. Poor linguistic command).

School attendance is one of the aspects of the child’s life that is most affected by their participation as a translator and which is of most concern to the adults in the community, due to its consequences for the child’s feelings, progress at school and maintaining the motivation to study: “*So I used to miss secondary school a lot , and I used to cry and I didn’t want to, I didn’t want to miss it because I loved going to school because I was very happy there and the teachers liked me a lot, and I cried because I didn’t want to go, but I had to because I was a girl.* […] *Boys, boys obey the father, but for him education is very important. Imagine, you are halfway through the year or in the second term and what do you do? You don’t have any motivation”* (II7. User. She translated when she was a child).

Long term concerns include the consequences of these barriers to the education of some of this country’s citizens, because there is great deal of emphasis on the future Spanish citizenship of these children: *“How many times does this child not go to school?* […] *Let’s count only the times with the doctor, apart from the others* […] *If you look at Spain now, there are many immigrant children who are… who are this country’s future, and work needs to be done in this area because they are the children of the future, the future of this country*” (II11. Mediator. Woman).

On an emotional level, the participants mention children’s embarrassment when translating what are considered adult subjects, such as sex and reproduction: “*She says that perhaps a small child cannot… for example, birth control and things like that, the child has a hard time, yes*” (FG9. Mixed. Poor linguistic command). And they worry about the impact that this early assumption of responsibilities can cause: “*They shouldn’t have to take responsibility, they are still too small. No, a 10-year-old girl doesn’t know about relationships, her mother’s sex life and all that…*” (FG9. Women. Mixed cultural affiliation and origin), and the anxiety that this direct involvement in healthcare issues may cause in children: “*Perhaps the child becomes worried, if they see that their mother is ill*” (FG9. Mixed. Poor linguistic command).

The inversion of the family hierarchy due to the power that child translators have over family members that depend on their help has been one of the effects identified that has a potential impact on the child’s development and family relations: “*Besides, what does that lead to, if the child has the information and the mother does not have it? It may lead to some power, the child having power, right?*” (II8. Mediator. Man).

Finally, showing solidarity with those who need help can have beneficial effects on the child’s self-esteem and well-being. This is suggested by the testimony from the adult acted as a translating minor: “*Yes, a lot of women told me how they felt, about coming from there, you understand? We chatted, while we went on the bus, we talked…* […]*. Yes, because* […] *I consider myself a good person, I can’t explain it, I like to be honest and I’m not proud, and these women can see that, that’s why many of them like me, because I’m simple.* […] *and they are grateful, because, really, you feel very frustrated when you feel ill, you see that your child is sick and don’t know how to explain this pain, you know?*” (II7. User. She translated when she was a child).

### Proposals for improvement

One proposal is collaboration between institutions and educational and health professionals to counter the lukewarm attitudes that are tolerant of the presence of children acting as translators during appointments. Some participants call for firm attitudes on the part of professionals, saying that they should prevent parents from using their children as translators. This proposal is based on the belief that people in need of translation using their children as interpreters will cease to do so when they believe that this is an unwavering requirement of the government: “*They need to agree, both the school and the doctor’s surgery and the hospitals about not accepting children* […] *because when they see that something is strict, the Moroccans comply with it, but if not…*” (II7. User. She translated when she was a child).

The need to provide a sufficient mediation/translation service is reiterated: “*I don’t know… it’s almost impossible. It is impossible, for the moment… there aren’t many resources and so far the service hasn’t been organized very well and we work… there are only a few mediators, and we work in the surgeries in Tarragona and we can’t meet the demand and yes, children continue to accompany their parents*” (II10. Mediator. Women), and for it to be organized proactively: “*The problem is that I think that not everyone who doesn’t speak the language knows that there is a mediator, do you understand? If they knew, it wouldn’t be necessary for them to take their children. To do that you’d need to do a talk at the school, one in the surgery or when they go to their Catalan course, explain that there is a mediator and how they can ask for one, you know*?” (II7. User. She translated when she was a child).

It is necessary to promote language learning among Maghrebi users: *“The solution? Well … first, in the short term, mediation, the health worker… In the long run, the women should study. Study, study, study! That’s the most important thing*” (II9. Mediator. Woman), although there were calls for a change of approach in this type of activities, which have been described as overly theoretical, repetitive and unhelpful for real communication needs: “*Yes, they needed to learn the language, but not in a way that … er … come here… fifty women and always with the same level of the course: this year level A, and the next year, and the next one, the next one… And the woman says: “every year I go there I hear the same thing”* (II12. Mediator. Woman).

Finally, mention is made of the need to motivate Maghrebi women to overcome their inertia when remaining at home and relating almost exclusively to their own community. There is a suggestion that necessity is a good incentive for giving up their passivity: “*They don’t want to study because they are all right… they’re all right, they’re fine. I don’t think it’s because their husbands won’t let them, it’s because of her, because she’s fine, she’s at home watching TV, she’s with her friends from her culture … she doesn’t need … to go shopping? Her husband. To translate? Her son… in other words, she has it all sorted out*.” (II9. Mediator. Woman).

## Discussion

There is little existing literature on the subject and that which exists is mainly descriptive. No studies were found that provided proposals for improving or regulating the presence of child translators in health care contexts.

The study revealed that Maghrebi adults attribute the use of children as translators in health services to the underfunding of translation resources and prioritization of adults’ employment over children’s education. The use of minors as translators is seen as a convenient solution to the community’s communication problems, although it is considered unreliable, affects the children’s rights and perpetuates the adults’ dependence. The attitude of healthcare professionals to the phenomenon studied is perceived as diverse and ranges from acceptance without any ethical questioning to concern about effects of the practice studied on the minor. The solutions proposed are an institutional change of attitude regarding the use of child translators, improved organization of translation resources and their adaptation to real needs, as well as a change in the focus of language training initiatives aimed at the community.

The lack of formal translation resources and the need to improve their organization has previously been highlighted by other studies in the field, which state that despite the variety of resources available, there is a need to optimize their operation, adjust the extent of resources to meet real needs and promote the service among professionals and users [3,26].

The priority given to adults’ employment obligations over children’s educational needs is in contrast to the concern expressed by adults about the educational interference caused by the minor’s participation in translation. The adults interviewed justify this ambivalence by the need to earn a living, and identify being absent from work for health reasons with a loss of income. Although accompanying relatives when visiting health services during working hours is not penalized financially in Spain, the prevention expressed by the information providers in this respect could be due to some degree of perceived discrimination in employment. Agudelo *et al*. [27] mention experiences of employment discrimination aimed at immigrants: racism, abuse and job insecurity are part of this type of experience, and have been identified as key factors in access to health services. In this case, recognition of immigrants’ employment and social rights would reduce cases of children being used as translators and the consequences resulting therefrom.

The shortcomings of translation guided by children and its impact on the lives of the children concerned have been described in detail in the literature [11,16-19] and are consistent with those in this study. There is agreement in both cases as regards mentioning the following problems as major issues: the risks of basing clinical decisions on an unreliable translation, the emotional impact that the task may have on the child, the inversion of the family hierarchy and the displacement of activities that should be carried out by children so that they can act as translators. However, subtle differences are apparent as regards the importance given to children’s school absenteeism. While the literature consulted contains few studies concerned with school absenteeism or mentions it anecdotally, in this study school absenteeism is at the heart of many of the arguments about the harmful effects of using children as translators, and which in turn inspires many of the proposals for solution. Cohen [16] mentions it as a minor and unavoidable problem in his work with general practitioners, if the aim is to provide care for adults with a poor command of the language. The minors who provided their testimony in the study by Free [18] also mention the difficulty of reconciling acting as a translator with school attendance, although they do not complain about it to a great extent. Finally, Giordano [15] also mentions this phenomenon in his review study. The differences between the results of previous studies and those in this study may be due to the nature of the information providers. In the publications reviewed, the adult information providers do not belong to the affected group, while the adults are participating in this research do. This would explain the minimization of the importance of education for children “of others.” Furthermore, the adults that express this attitude are healthcare professionals whose priority is to receive help in translation. As for the testimony of minors in the study by Free [18] it is to a certain extent logical that children do not place too much importance on going to school because they may lack the sense of usefulness that adults see in it. There were no children talking about their experience in our study, although there is indirect evidence from parents, mediators and an adult who used to be a translating minor. Obviously, the perspective is not the same when seen from an adult or a child’s viewpoint; in any event, the participants in this study regret the loss for children involved in regularly being absent from school in order to provide accompaniment for health service appointments. However, in accordance with the results of the study by Free [18], when they are faced with the dilemma, the concern for the adults’ health and the improved self-esteem they experience as a result of the help prevails.

The case study by Jacobs [23] shows the extreme effects of involving children in tasks that exceed their stage of development and warns of the potential risks to children’s physical and mental health if they are used as translators in delicate situations. None of the participants in this study have witnessed children passing on bad news. Although these circumstances cannot be ruled out, professionals appear to take it into consideration and assess the harm that this could cause to the child, especially when there are formal and informal resources that can be used when there is the possibility that the child’s health may be affected.

The adults in the community–users and mediators–believe that there is no unanimous attitude among professionals, some of whom accept child translators because of the immediate need for translation for the appointment, without considering the future consequences for the children acting as translators, the Maghrebi community and society as a whole. However, the fact is that although they try to minimize the consequences of this practice, Maghrebi adults cooperate with it. The attitude of the mediators interviewed, as both health professionals and Maghrebis, shows a concern for the future effects of using children as translators, especially in terms of equal opportunities and coexistence between citizens of different origins. As stated in the introduction to this article, many authors disapprove of translation provided by minors, although the reasons for this rejection do not include the long-term consequences that this practice may have for both the affected community and society as a whole. Concerns about the overall impact of interfering with the right to education of part of society should provide political arguments so that interventions specifically targeted at the empowerment of a community can be seen as beneficial to society as a whole.

The proposals for improvements made by the participants include a request for healthcare and education professionals to be more intransigent as regards the use of children as translators. One requirement of this type requires the provision of translation resources that meet real needs. According to the comments by the mediators interviewed and the information provided by a service evaluation study, intercultural mediation services in health in the area are not stable and do not meet the entire demand [26]; moreover, the current economic crisis suggests that it is difficult to adjust them appropriately. Although it has been indicated that providing interpreter services is a financially viable method for enhancing delivery of health care to patients with language barriers [28], there are no studies to support this finding in Spain. The addition of mediators to the health team and the organization of the service based on a proactive approach are solutions without any added financial cost that could optimize the performance of the resources available.

The long-term solution lies not only in providing formal translation resources and the firmness of the environment, but also actions to promote and facilitate the autonomy and empowerment of adults who are dependent on translation. There was criticism of the lack of motivation among to initiate language training Maghrebi users, and it was suggested that the best way to motivate them to learn the language lies in making them feel the need to do so. On the other hand, the existing training initiatives were criticized by some as not being stimulating or useful for dealing with everyday challenges practically. The challenge therefore lies in making them more appealing, and changing the perception of usefulness of language training initiatives among Maghrebi users with a poor linguistic command of Spanish. One intervention that could be useful in both respects would be to minimize classroom training and use real-life situations as formal language learning environments. This move away from the classroom should ideally include support in learning how to deal with everyday situations and the exchange of experiences between immigrants and natives. This type of focus would lead to the implementation of the knowledge learned in class, the sedimentation of learning, practical training for specific situations, formal access to knowledge about the health system and contact with the host society. It would be naive to think that it would be easy to implement this type of initiative. First, it will be necessary to overcome the inertia of some users to move beyond the small but secure circle of their immediate environment, and subsequently, to overcome possible resistance from the Spaniards to getting to know Maghrebi users.

The use of child translators in appointments can be considered a temporary and unsatisfactory solution to the communication problems experienced by the Maghrebi community. The real solution requires effort on both sides. It requires a range of resources to be made available to the user, firmness by professionals in defending equal opportunities for children who are deprived of education, and effort by the users in giving up the passivity of incapacitating help and the convenience of dependency.

One of the limitations of this study is the lack of focus groups segmented according to the information providers’ legal status. The participation of individuals who had spent less than one year in Spain, and of two adults without any access to health services may alleviate this deficiency to a certain extent, but there is a risk of diluting the discourse of people with difficulties in accessing the health services within other dominant discourses. Moreover, the fact it was impossible to organize an entirely male focus group prevents access to the male group attitude, and although there were several male information providers, it was not possible to determine whether their statements differ from those that would have been obtained in the focus group.

Since education is a basic determinant of health and a key factor in empowerment initiatives [29], quantitative studies that provide objective data on the presence of Maghrebi children as translators in the health services are necessary, as well as other qualitative studies about the effects of interference in the education of Maghrebi children, in terms of both their welfare and development and for the future coexistence between citizens of different origins.

## Conclusions

The main contribution of this paper, which deals with immigrants who speak languages with few speakers in the host society, is to provide proposals that reconcile the adults’ right to health and work with the rights of the children who are required to act as their translators. These proposals could be extrapolated to other contexts where there is universal and free access to health and education services and where there is insufficient provision of public translation services.

If the rights of Maghrebi adults are to be reconciled with the rights of children who act as their translators in health care contexts, consideration needs to be given to coordinating the efforts of health and educational institutions, changing the organization and the provision of translation resources, and ensuring that immigrants have the same employment rights as Spanish nationals.

## Abbreviations

PHC: Primary healthcare centre; CSE: Compulsory secondary education; FG: Focus group; II: Individual interview.

## Competing interests

The authors declare they have no competing interests.

## Author’s contributions

(LR-R) conceived and designed the study, and gathered, analyzed and interpreted the data; she wrote the article and is the main author. (ARB), (IdMF) and (MMVG) collaborated in the data analysis and the critical revision of the article, and made substantial contributions to the final draft. All authors read and approved the final manuscript.

## Supplementary Material

Additional file 1: Table S1 Key issues in the interviews/focus group sessions investigated in the entire project and trigger questions on the use of child translators in the health services. Click here for file
