# Supplementary material for: Maghrebi minors as translators in health services in Tarragona (Spain): a qualitative study of the discourse of the Maghrebi adults
Source: Global Health. 2014 May 7;10:31. doi: 10.1186/1744-8603-10-31 (PMC4024186; doi:10.1186/1744-8603-10-31)
Supplement: Additional file 1: Table S1 — Key issues in the interviews/focus group sessions investigated in the entire project and trigger questions on the use of child translators in the health services. [file 1744-8603-10-31-S1.pdf]

Additional file 1: TABLE S1 KEY ISSUES IN THE INTERVIEWS/FOCUS GROUP SESSIONS INVESTIGATED IN THE ENTIRE PROJECT AND TRIGGER QUESTIONS ON THE USE OF CHILD TRANSLATORS IN THE HEALTH SERVICES

	Key issues	Trigger questions
**Individual interviews and focus groups of users**	Overall opinion	Have you used school age children to help as translators? Under what circumstances? Do you know of other people who have done this? What is your opinion of the use of children as translators? What do you think the professionals think of it? How does it affect the people involved? And what about health interaction? Any suggestions for minimizing the use of children as translators?
	Health system	
	Development of health interaction	
	Treatment by professionals	
	Communication with professionals	
	Use of minors as translators →	
**Individual interview with cultural health mediators**	Role of mediators	Do you know of users who use school age children as translators? Why do you think that this happens? What is your opinion of the use of children as translators? What do you think the professionals think of it? How does it affect the people involved? And what about health interaction? Any suggestions for minimizing the use of children as translators?
	Maghrebi user's health needs	
	Communication with professionals	
	Use of minors as translators →	
